# Recent Advances in Multi-Drug Resistance (MDR) Efflux Pump Inhibitors of Gram-Positive Bacteria *S. aureus*

**DOI:** 10.3390/antibiotics2010028

**Published:** 2013-02-05

**Authors:** Jadwiga Handzlik, Anna Matys, Katarzyna Kieć-Kononowicz

**Affiliations:** Department of Technology and Biotechnology of Drugs, Faculty of Pharmacy, Jagiellonian University-Medical College / ul. Medyczna 9, 31-688 Cracow, Poland; E-Mails: anna.dymek@uj.edu.pl (A.M.); mfkonono@cyf-kr.edu.pl (K.K.-K.)

**Keywords:** bacterial multidrug resistance, MDR, efflux pump inhibitors, EPIs, NorA

## Abstract

The paper focuses on recent achievements in the search for new chemical compounds able to inhibit multidrug resistance (MDR) mechanisms in Gram-positive pathogens. An analysis of the results of the search for new efflux pump inhibitors (EPIs) for Gram-positive bacteria, which have been performed over the last decade, indicates that almost all efforts are focused on the NorA (MFS) efflux pump in *S. aureus*. Considering the chemical structures of the NorA EPIs that have been identified, it can be observed that the most active agents belong to the families of compounds possessing conjugated double bonds, e.g., chalcones, piperine-like compounds, N-cinnamoylphenalkylamides or citral amide derivatives. Indole-, dihydronaphthyl-, 2-chloro-5-bromo-phenyl- or piperidine moieties seem to be profitable for the EPI properties, as well. These results, together with an increasing knowledge about a variety of efflux pumps that are involved in MDR of Gram-positive pathogens underline that further search for new EPIs should pay more attention to develop MDR efflux protein targets, including SMR, MATE, ABC or other members of the MFS family.

## 1. Introduction

As a consequence of the intense fight against infections, bacteria have evolved through numerous defenses against antimicrobial agents [1]. The main mechanisms whereby the bacteria develop resistance to antimicrobial agents include enzymatic inactivation [2,3], modification of the drug target(s) [3,4], and reduction of intracellular drug concentration by changes in membrane permeability [3,5] or by the overexpression of efflux pumps [3,6]. With respect to efflux pumps, they provide a self-defense mechanism by which antibiotics are actively removed from the cell. For antibacterials, this results in sublethal drug concentrations at the active site that in turn may predispose the organism to the development of high-level target-based resistance [3,7]. Therefore, efflux pumps are viable antibacterial targets and identification and development of potent efflux pump inhibitors is a promising and valid strategy [3,8] which can restore the susceptibility of resistant strains to antibacterial agents that are substrates of efflux pumps [3,9]. The combination of a resistance inhibitor with an antibiotic has already proven its efficacy with the clavulanic acid (inhibitor of beta-lactamase)/amoxicillin association [10]. Predominantly, the world search for new tools to combat multidrug resistance (MDR) among bacterial pathogens is concentrated on Gram-negative bacteria aspects [11,12,13,14,15,16,17,18] because of their more complicated MDR mechanisms due to their double-membrane cells, which allow the expression of a tripartite efflux pump system such as AcrA/AcrB/TolC in *Enterobacteriaceae* [19,20,21] or MexA/ MexB/OprM in *Pseudomonas aeruginosa* [22,23,24]. Although the 3D-structures of protein components of the tripartite efflux pumps have been identified experimentally [25], which should simplify studies on inhibitor-binding pockets, the knowledge about the pump-inhibitor interactions is still not sufficient to involve the “protein-ligand drug design” approach in the search for new EPIs for Gram-negative pathogens. Results of recent microbiological- and medicinal chemistry studies allowed us to identify several chemical families of compounds inhibiting tripartite MFP/RND/OMF pump action [12,13,16,25] but it is hard to find a good pharmacophore model resulting from the studies that could be applicable in further design of new potent EPIs.

Indeed, various lines of evidence [1,3,10,26,27,28,29,30,31,32,33,34,35,36,37,38] have indicated a significant development of medicinal chemistry tools useful in the search for efflux pump inhibitors for Gram-positive pathogens. As multidrug resistant Gram-positive bacteria have been and still are a current therapeutic problem, it is of great importance to analyze the recent progress in the search for new tools to combat it. Thus, this paper focuses on recent achievements in the search for new chemical compounds able to inhibit MDR mechanisms in Gram-positive pathogens. 

## 2. Efflux Pumps in Gram Positive Bacteria

Efflux pumps in Gram-positive bacteria belong to four unrelated families (Table 1): MFS (major facilitator superfamily), SMR (small multidrug resistance), ABC (ATP-binding cassette) and MATE (Multidrug And Toxic Compound Extrusion) [9,39,40,41,42,43,44,45,46,47,48,49,50,51,52,53,54]. 

MFS transporters are typically composed of approx. 400 amino acids that are putatively arranged into 12 membrane-spanning helices, with a large cytoplasmic loop between helices six and seven [39,55,56]. The examples of MFS efflux pumps in Gram-positive bacteria are NorA, NorB, MdeA, Tet38 (*Staphylococcus aureus*), LmrB, Bmr, Bmr3, Blt (*Bacillus subtilis*), MefA (*Streptococcus pyogenes*), MefE (*Streptococcus pneumoniae*) or CmlR *(Streptococcus coelicor)* [39,55,57,58,59]. SMR transporters consist of approx. 110 amino acids and contain four transmembrane helices. 

**Table 1:** Efflux pumps in Gram positive bacteria and their role in antibiotics transport.

Bacterial strain	Transport protein family	Efflux pump	Substrates
*Staphylococcus aureus*	MFS [9,41,42,43,44]	NorA	NOR, CPX
		NorB	NOR, CPX, SPX
		MdeA	Macrolides
	MATE [45]	Tet38	tetracyclines
		MepA	FQ, glycylcyclines
	SMR [39,60,61]	Smr, QacG, QacH	-
*Staphylococcus spp*	MFS [9,46,47]	Mef(A)	Macrolides
	ABC [47,48,49]	MsrA	Macrolides, type B streptogramins
*Staphylococcus haemolyticus*	MFS [42]	MdeA	Macrolides, lincosamides type A streptogramins
*Staphylococcus lentus*	[54]	FexA	-
*Streptomyces coelicolor*	MFS [38]	CmlR1, CmlR2	Chloramphenicol
*Streptomyces spp*	MFS [50]	Cml, Cmlv, Cmr, Cmx, CmA	-
*Streptococcus spp*	MFS [9,45,47]	Mef(A)	Macrolides
*Streptococcus pneumoniae*	ABC [51]	Msr(D)	Macrolides, ketolides
	MFS [52]	PmrA	NOR, CPX
*Clostridium difficile*	MFS [53]	Cme	Erythromycin
	MATE [45]	CdeA	FQ
*Bacillus subtilis*	MFS [9,46]	LmrB	Lincosamides
		Bmr, Bmr3, Blt	FQ
*Bacillus glutamicum*	MFS [9,46]	LmrB	Lincosamides

NOR: norfloxacin; CPX: ciprofloxacin; SPX: sparfloxacin; FQ: flouroquinolones.

Owing to the small sizes of the proteins that belong to this family, they probably function as oligomeric complexes [39,59]. The examples of SMR efflux pumps in Gram-positive bacteria are EbrAB (*Bacillus subtilis*) or Smr, QacG, QacH *(Staphylococcus aureus)* [39,60,61]. MATE efflux proteins consist of 400–700 amino acids that form 12 transmembrane helices. All proteins of the MATE family exhibit almost 40% identity of their amino acid sequence. All genes that encode MATE proteins are derived from the same gene which was subsequently duplicated. An example of MATE efflux pump in Gram-positive bacteria is MepA protein found in *Staphylococcus aureus* [62,63]. MFS, SMR and MATE transporters use a transmembrane proton gradient as the driving force for transport [39,62,63,64,65]. The minimal structural organization of an ABC transporter includes the presence of four domains, *i.e*., two nucleotide binding domains (NBDs) and two transmembrane permease domains (TMDs). The TMDs usually consist of six transmembrane α-helices and form homo or heterodimers. Two NBDs bind ATP in the cytoplasmic side and cooperate with transmembrane domains [39,65,66].

The feature which distinguishes ABC transporters from the remaining families is the energy source for active extrusion of drugs, as it comes from the hydrolysis of ATP. Binding and hydrolysis of ATP triggers conformational changes in the transporter’s structure, which enable export of substrates [39,67]. The examples of ABC efflux pumps in Gram-positive bacteria are LmrA *(Lactococcus lactis)* or Rv1217c–Rv1218c (*Mycobacterium tuberculosis*) [39,55,68].

Multidrug resistance efflux pumps are recognized as an important component of resistance in both Gram-positive and Gram-negative bacteria. Some bacterial efflux pumps may be selective for one substrate or transport antibiotics of different classes, conferring a multiple drug resistance (MDR) phenotype. Efflux pumps inhibitors (EPIs) are promising therapeutic agents, as they should restore the activity of standard antibiotics. The efflux pump inhibitor-antibiotic combination is expected to increase the intracellular concentration of antibiotics that are expelled by efflux pumps, decrease the intrinsic bacterial resistance to antibiotics, reverse the acquired resistance associated with efflux pumps overexpression, and reduce the frequency of the emergence of resistant mutant strains [69].

Bypassing efflux pump activity may be achieved through a variety of different approaches: (1) by modifying the chemical design of previous antibiotics to reduce their respective affinity for binding sites and cavities located inside the pump transporter; (2) by increasing the influx of antibiotics, using membrane permeabilizers that subsequently increase the intracellular concentration of drugs; (3) by down-regulating the expression of efflux pump genes and/or decreasing the level of active efflux complexes in the bacterial envelope; (4) by collapsing the energy required to support the drug transport; (5) by inhibiting the functional assembly of efflux pump components; (6) by inserting a carefully-designed molecular plug inside the membrane channels responsible of antibiotic transport (inside the pump cavities or inside the exit channel component) or (7) by generating a dynamic competition, between a decoy- substrate and the antibiotic, during transport flux inside the pump [12].

## 3. Current Approaches in Search for EPIs in Gram Positive Bacteria

The methicillin-resistant *Staphylococcus aureus* (MRSA) is a major multidrug resistant Gram-positive bacteria that is a main cause of healthcare-associated infections (HAIs) resulting in a high death rate. MRSA is able to acquire resistance to various antibiotics, including tetracyclines, aminoglycosides and flouroquinolones. Studies on MDR efflux mechanisms in *S. aureus* indicated that NorA is predominant protein efflux pump [3]. For these two reasons, NorA in *S. aureus* is a frequently studied efflux pump as well as being the main protein target in the search for efflux pump inhibitors in the case of Gram-positive bacteria. 

Recent decades have seen the production of a number of new chemical compounds belonging to various chemical families, which were investigated on their NorA EPI properties [3,10,26,27,28,29,30,31,32,33,34,35,36,37,38,70]. In the studies, an examination of the new compounds on their EPI properties have predominantly been based on: (1) a comparison of antibiotics efficacy in the presence- to that in the absence of the tested compound in the strain over-producing efflux pump and/or (2) the assays of inhibition of a substrate-efflux, mediated by the efflux pump, at various concentrations of the tested compound. In both types of assays, *S. aureus* SA 1199B was the most often used strain over-producing NorA efflux pump, and the wild strain *S. aureus* SA 1199 was involved as a reference one. Ciprofloxacin (CPX) is described as the most often used antibiotic, and ethidium bromide (EtBr) as the main reference substrate of NorA applied in the (real-time) efflux assays.

### 3.1. Plant-Derived NorA EPIs and Their Chemical Modifications

The role of phytochemistry in search for compounds inhibiting NorA of S.aureus is significant as it is reported to be an extremely varied series of plant-derived EPIs displaying different chemical properties, including flavones, isoflavones, acylated glycosides, porphyrin phaeophorbide A or kaempferol rhamnoside [34,70].

**Figure 1:**
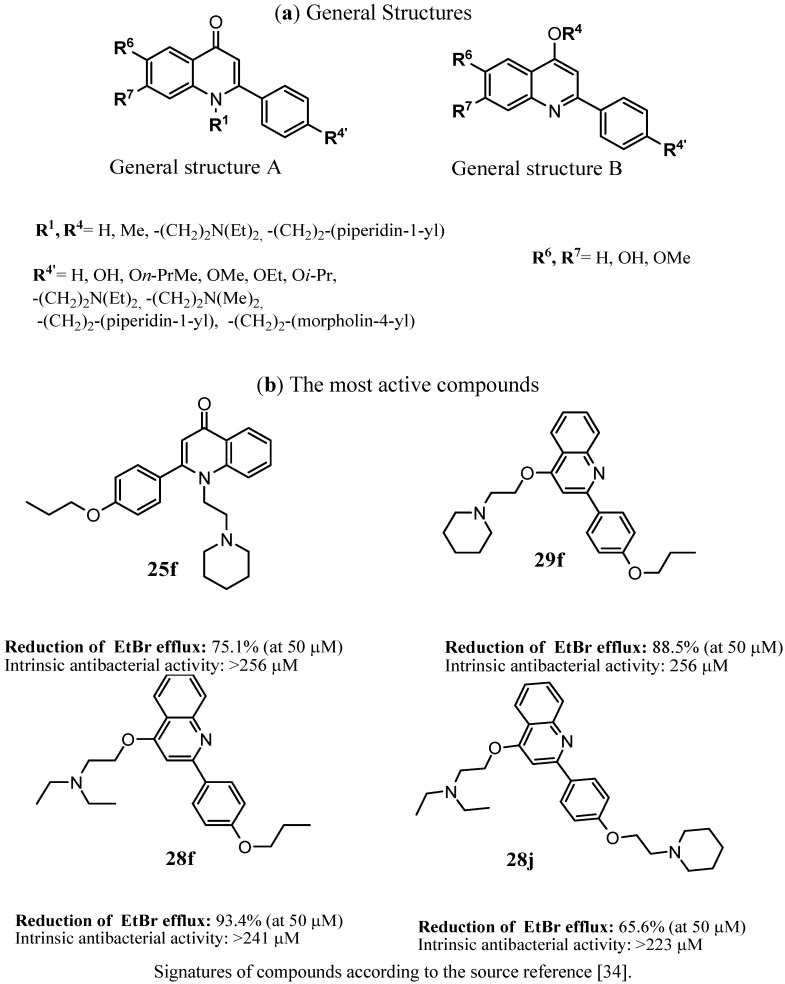
The most active inhibitors of the *S. aureus* NorA efflux pump among 2-(4-propoxyphenyl)quinoline [34].

Chemical structures of the natural EPIs have been a good starting point for further modifications to search for new inhibitors with higher potency and better pharmacological profile. In this context, Sabatini *et al*. [34] described the synthesis of a series of 2-(4-propoxyphenyl)quinoline derivatives, that was designed on the basis of natural flavones nucleus (Figure 1). The compounds were investigated on their EPIs action in the ethidium bromide (EtBr) inhibition assays on strain *S. aureus* SA-1199B over-expressing norA. The most active compounds **25f**, **28f**, **28j** and **29f** displayed more than 65% inhibition of EtBr at their 50 µm concentration.

**Figure 2:**
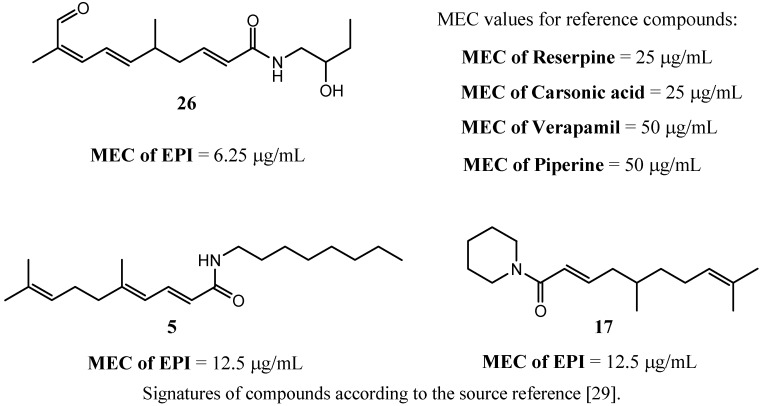
Citral amide derivatives that potentiate MIC of ciprofloxacin on *S. aureus* 1199. The most active compounds found within the assays [29]. MEC value describes minimal effective concentration of the tested compounds.

Thota *et al*. [29] described a series of citral derived amides that have been obtained from monoterpene citral or citronellal (Figure 2).

The compounds were investigated on their abilities to decrease minimal inhibitory concentration of ciprofloxacin in the strains of *S. aureus* over-producing NorA pump. The EPI-action of the compounds was expressed as MEC-value (minimal dose of the tested compound that caused a decrease in the MIC of ciprofloxacin). As reference compounds, reserpine, carsonic acid, verapamil and piperine were used. The most active compound, 2,6,8-trienoic acid amide derivative **26** improved antimicrobial action of ciprofloxacin at the compound concentration 4–8 fold lower than that of reference inhibitors. Dienoic acid piperidide derivative (**17**) and 2,4,8-trienoic derivative of octylamide (**5**) were active at their doses 2–4 fold lower than those of reference EPIs (Figure 2) [29].

### 3.2. Chalcones and Alkenamide Inhibitors of the NorA in S. aureus

**Figure 3:**
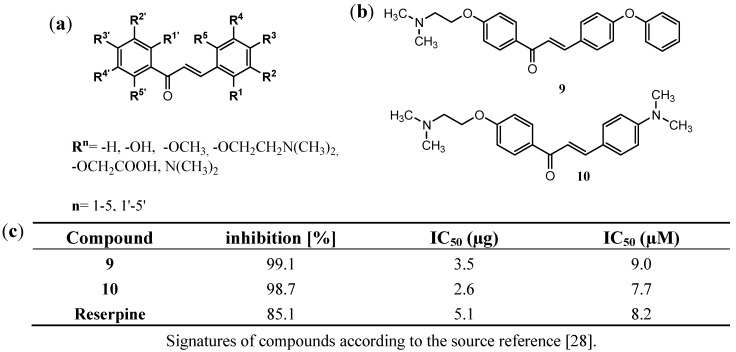
Chalcone inhibitors of the NorA efflux pump in *S. aureus*; (**a**) the general structure of the chalcone EPIs; (**b**) the most active inhibitors found in the real-time efflux (RTE) assay; (**c**) Inhibition of EtBr efflux in SA1199B at compounds concentration of 20 µg/mL for the most active chalcones **9** and **10** [28].

The newest work of Holler *et al*. [28] focuses on a series of 117 chalcone derivatives modified at each position of both phenyl rings (Figure 3a). The compounds were tested on their EPI activity against NorA mediated ethidium bromide efflux in real-time efflux assay (RTE) in the strain SA1199B. Five active compounds were found. Among them two N,N-dimethylaminoethoxyphenyl derivatives **9** and **10** (Figure 3b) were equipotent to reserpine, that was used as reference EPI in the studies. The compounds tested at their concentration of 20 µg/mL displayed almost total inhibition of EtBr efflux (Figure 3c).

**Figure 4:**
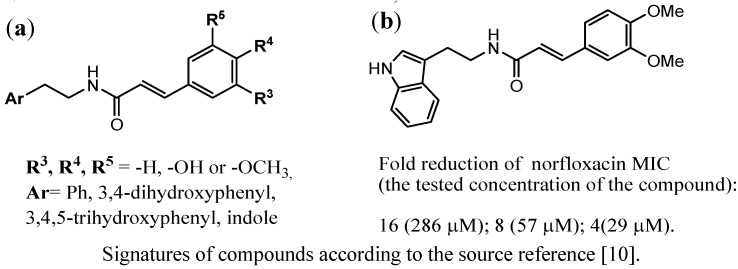
N-cinnamoylphenalkylamide derivatives investigated according to their efflux pump inhibitors (EPIs) action in the strain over-producing NorA pump; (**a**) general structures of the N-cinnamoylphenalkylamides; (**b**) the structure of the most active inhibitor [10].

Michalet *et al*. [10] performed studies among N-cinnamoylphenalkylamide derivatives with (un)substituted phenyl or indole at one terminated fragment and (un)substituted phenyl at the opposite terminate fragment (Figure 4a).

In the case of both aromatic fragments electrodonated substituents were considered. The most active compound (Figure 4b), possessing indole moiety and 3,4-dimethoxycinnamoyl fragment, caused a 4-fold reduction of norfloxacin MIC at the lowest concentration of 29 µM.

**Figure 5:**
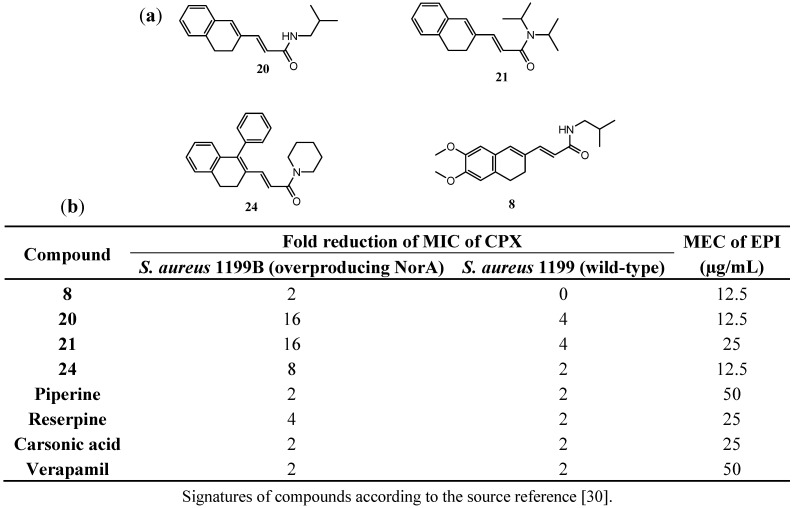
Dihydronaphthalene inhibitors of the NorA efflux pump in *S. aureus* [30]. (**a**) Structures of the most active compounds (**20**, **21**, **24** and **8**); (**b**) Abilities of the most active compounds to potentiate antimicrobial action of ciprofloxacin (CPX) in comparison to reference compounds.

Compounds with similar topology of conjugated double bonds were obtained and investigated by Thota *et al*. [30], who found new EPIs among derivatives of 3,4-dihydronaphth-2-yl-propenoic acid (Figure 5). The compounds were examined according to their ability to improve the efficacy of ciprofloxacine in MDR strains of *S. aureus* (1199B and 1199). The most active compounds included chemical moieties of isobutylamide (**8**, **20**), diisopropylamide (**21**) and piperidide (**24**). The compounds were able to reduce MIC of ciprofloxacine (CPX) in 2–16-fold at their concentrations 1–4 fold lower than those of reference EPI (Figure 5). The simple structure of 3-(3,4-dihydronaphth-2-yl)-propenoic acid isobutyl amide (**20**) was the most promising one, which caused a 16-fold reduction of MIC of CPX in strain 1199B and 4-fold in the case of 1199 strain. Its MEC value (12.5 µg/mL) was 4-fold lower than that of piperine and verapamil and 2-fold lower than that of reserpine and carsonic acid.

### 3.3. Piperine EPIs against NorA in S. aureus

**Figure 6:**
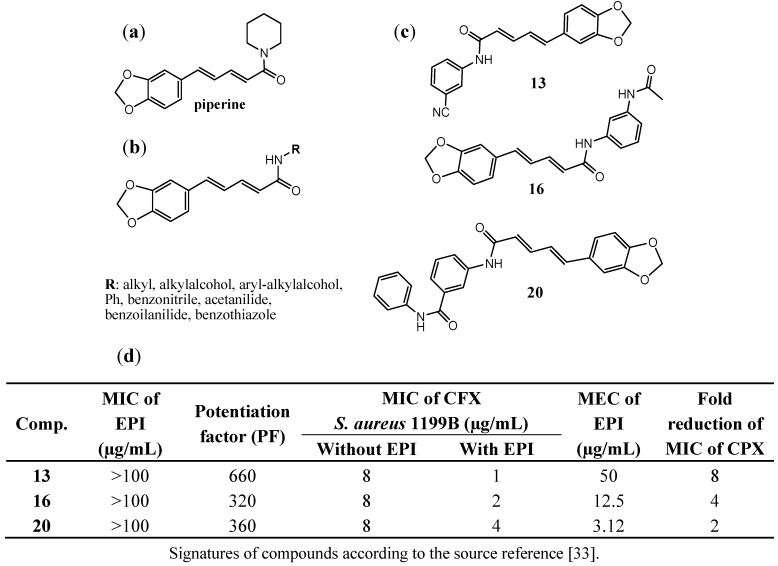
Piperine analogs that decrease MIC of ciprofloxacin in MDR *S. aureus* strain over-producing NorA (1199B); (**a**) structure of piperine; (**b**) the general structure of the tested compounds; (**c**) the most active compounds **13**, **16** and **20**; (**d**) Abilities of compounds **13**, **16**, **20** to increase efficacy of ciprofloxacin [33].

Piperine (Figure 6a) is an alkaloid that was isolated from the fruits of *Piper nigrum* by H.C. Orsted in 1819. This natural compound displays properties of the inhibitors of some proteins important for metabolism and xenobiotics transport and it is used as a reference inhibitor in studies on new efflux pump inhibitors [29,30]. On the basis of the inhibitor properties of piperine, Nargotra *et al*. [33] designed and synthesized a series of piperine analogs, in which piperidine moiety was modified by replacement with other amines (Figure 6b). The new compounds were investigated according to their ability to reduce MIC of ciprofloxacin in *S. aureus* 1199B strain using eight different concentrations of the investigated compounds (0–50 µg/mL). Potentiation factor (PF) was calculated to express the potentiation of activity of ciprofloxacin in the presence of the tested new EPIs, reflected in the reduced MIC of combination compared to that of ciprofloxacin alone (Fig 6d). Three of the most promising EPIs were found (Figure 6c) in which the piperidine fragment of piperine was replaced with 3-aminobenzonitrile (**13**), N-(3-aminophenyl)acetamide (**16**) or 3-amino-N-phenylbenzamide (**20**). The highest potentiation factor was observed for the benzonitrile amide derivative (**13)**, whereas the 3-amino-N-phenylbenzamide derivative (**20**) displayed EPIs activity at the lowest concentration with its MEC value of 3.12 µg/mL (Figure 6d). 

The series of compounds was used in quantitative structure-activity relationship studies (QSAR) to evaluate QSAR parameters that are responsible for NorA EPIs. In the QSAR studies, the authors considered seven categories of descriptors as follows: energy-state indices, electronic, information content, spatial, structural, thermodynamic and topological ones. On the basis of the obtained QSAR model, the descriptors of the partial negative surface area (Jurs-PNSA-1) and area of the molecular shadow in the XZ plane (Shadow_XZ) were identified as the most important parameters that contributed to the potentiation of EPI activity. An increase of Jurs-PNSA-1 is connected with the introduction of a polar group into the structure of a modified EPI, which is able to increase the partial negative surface area of the compound. This kind of modifications is postulated to be profitable for NorA efflux pump inhibitory properties. Similarly, changes of the placement of substitutuent(s) (ortho- meta- or para), which increase Shadow_XZ parameter, are profitable for the activity. Furthermore, the parameter of heat of formation (Hf) was demonstrated as a factor important for NorA inhibitory properties in *S. aureus*.

### 3.4. Indoles as Inhibitors of the NorA of S. aureus

**Figure 7:**
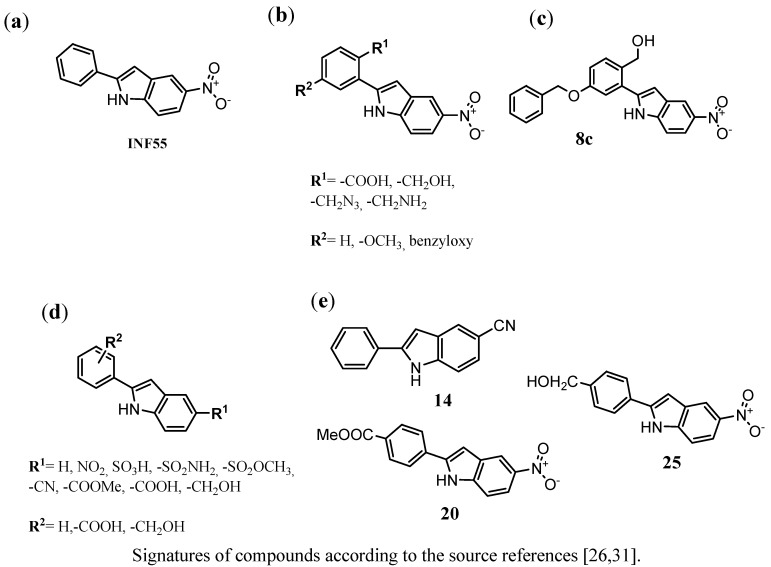
Derivatives or 2-aryl-1H-indoles that display NorA MDR inhibitory activity on *S. aureus* strains (K1758, 8325-4 and K2361); (**a**) lead structure **INF55**; (**b**) the general structure of the chemical group described by Samosorn *et al*. [31]; (**c**) the most active compound (**8a**) identified within the studies [31]; (**d**) the general structure of the chemical group described by Ambrus *et al*. [26]; (**e**) the most promising indoles-EPIs (**14**, **20** and **25**) identified [26].

Compound **INF55**, 5-nitro-2-phenyl-1H-indole (Figure 7a), was one of the first identified indole EPIs that was capable of producing 4-fold increase susceptibility of *S. aureus* to ciprofloxacin at its concentration of 1.5 g/mL. 

### 3.5. Other Chemical Groups of NorA EPIs

**Figure 8:**
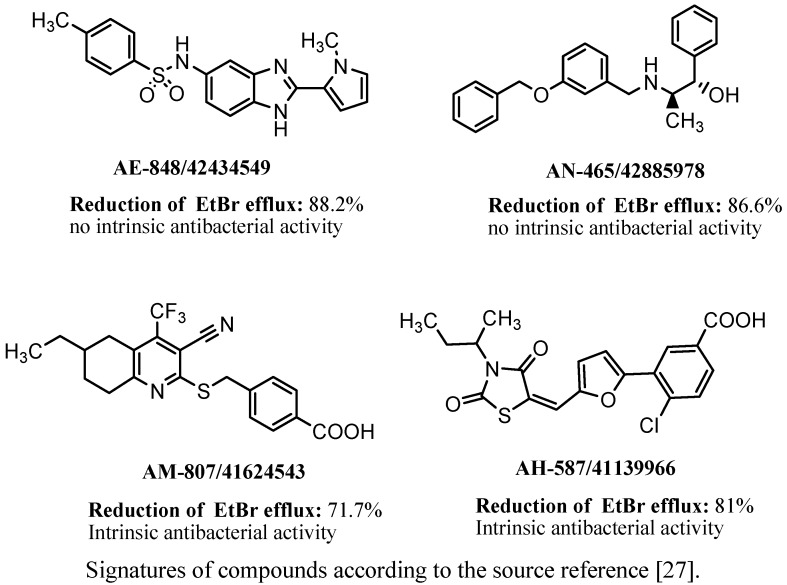
Four NorA EPIs found by the use of virtual screening and their EPIs potency to inhibit EtBr efflux in SA1199B strain [27].

Brincat *et al*. [27] identified four novel inhibitors of the NorA efflux pump of *S. aureus* belonging to different chemical groups. Structures of the compounds were discovered on the basis of virtual screening that involved FLAP procedure and new methodology using GRID force field descriptors. The compounds created within the ligand-based design were evaluated on their NorA EPI properties *in silico*. The structures identified as active *in silico* were then synthesized and evaluated experimentally on their EtBr efflux inhibition in *S. aureus* strain SA-1199B as well as on their ability to potentiate the antibacterial effect of ciprofloxacin. In this group, the bezimidazole derivative **AE-848/42434549** as well as the benzyloxybezylamine derivative **AN-465/42885978** (Figure 8) demonstrated the highest EPIs properties as both of the compounds significantly inhibited EtBr efflux and did not display intrinsic antibacterial activities. Two other compounds, pyridine- and rhodanine derivatives (Figure 8), demonstrated significant inhibitory properties but their intrinsic antibacterial action was significant as well.

### 3.6. PSSRI-Based EPIs of NorA and MepA in S. aureus

**Figure 9:**
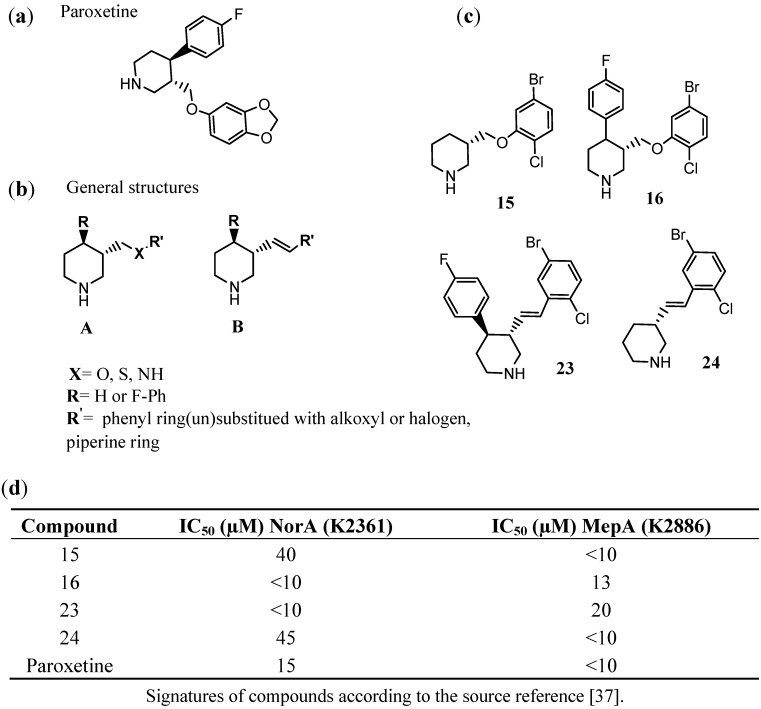
Phenylpiperidine selective serotonin reuptake inhibitor (PSSRI)-based EPIs of *S. aureus*. (**a**) Paroxetine; (**b**) general structures (A and B) of the investigated piperidine derivatives; (**c**) the most promising EPIs found within the assays (**15**, **16**, **23**, **24**) and their EPIs potency, expressed as IC_50_ values for inhibition of NorA (SA-K2361) or MepA (SA-K2886) in comparison to those of paroxetine [35,37].

Phenylpiperidine selective serotonin reuptake inhibitors (PSSRIs) are able to block the function of some MDR efflux pumps. Paroxetine (Figure 9a) was one of the first identified PSSRI that inhibits both NorA- (MFS family) and MepA (MATE)-efflux pumps. On the basis of this structure, further chemical modifications were performed (Figure 9b) to search for new and more potent EPIs [35,37]. German *et al*. [37] obtained interesting results when replaced benzo[d] [1,3] dioxole moiety of paroxetine with 2-chloro-5-bromo-phenyl fragment (**16**, Figure 9c) and exchanged the phenoxyl fragment (ether linker) into arylidene one (**23**, Figure 9c). Both compounds (**16**, **23**) displayed an increase of efflux inhibition of NorA comparing to that of paroxetine. A deletion of fluorophenyl substituent was also profitable to give selective EPIs properties for MepA (**15** and **24**, Figure 9c,d). 

### 3.7. Search for Inhibitors of Other Efflux Pumps of Gram-Positive Bacteria

Although most of the studies on efflux pump inhibitors for MDR Gram-positive bacteria are dedicated to NorA in *S. aureus*, a few lines of evidence are focused on other protein targets as well. Studies on MepA-inhibitors were mentioned in previous research [35,37]. Furthermore, Okandeji *et al*. [38] described C-capped dipeptides that inhibited chloramphenicol-specific efflux pumps, cmlR1 and cmlR2, in *Streptomyces coelicolor*, a strain of Gram-positive bacterium that is relative to the human pathogen *M. tuberculosis*.

## 4. Conclusions

An analysis of the results of the search for new efflux pump inhibitors for Gram-positive bacteria which have been performed for last decade indicates that almost all efforts are focused on the NorA efflux pump in *S. aureus*. Considering the chemical structures of the NorA EPIs that have been identified, it can be observed that the most active agents belong to the families of compounds possessing conjugated double bonds, e.g., chalcones, piperine-like compounds, N-cinnamoylphenalkylamides or citral amide derivatives. Indole-, dihydronaphthyl-, 2-chloro-5-bromo-phenyl- or piperidine moieties seem to be profitable for the EPI properties as well. 

To date, no inhibitors of bacterial efflux pumps have been licensed for use in the treatment of bacterial infections in human or veterinary settings, although research continues. As far as Gram-positive bacteria are concerned, none of the efflux pump inhibitors have entered clinical trials yet [69,71]. Nevertheless, in the last decade, studies on MDR Gram-positive EPIs allowed us to identify new active agents of NorA in *S. aureus* with EPI-potency significantly higher than that of reference inhibitors. The active compounds give a new hope for their future therapeutic usage as antibiotics “adjuvants” and should be a subject of wider investigations, including their pharmacokinetic properties and toxic effects. In particular, further studies should be concentrated on the influence of the NorA agents on eucariotic efflux- and influx transport proteins with a special consideration of human transporters belonging to the ABC or SLC families. As human proteins expelling toxic substances out of tissues display significant similarities to those involved in bacterial MDR, bacterial EPIs probably inhibit human detoxification simultaneously with the inhibition of bacterial MDR. This aspect requires thorough research for each active bacterial EPI that is considered as a future “adjuvant” of antibiotics.

In contrast to NorA inhibitors, a population of EPIs active against other MDR efflux proteins of Gram-positive bacteria, which have been found during the last decade, is very small. These results, together with an increasing knowledge about a variety of efflux pumps that are involved in MDR of Gram-positive pathogens, seem to be an important challenge for current medicinal chemistry. They underline the opinion that, in the future, the search for new EPIs should pay more attention to developing MDR efflux protein targets, including SMR, MATE, ABC or other members of the MFS family.
